# The prevalence and plasmid profile of non-typhoidal salmonellosis in children in Lagos metropolis, South-western Nigeria

**DOI:** 10.11604/pamj.2014.19.359.2322

**Published:** 2014-12-09

**Authors:** Ajoke Olutola Adagbada, Akitoye Olusegun Coker, Stella Ifeanyi Smith, Solayide Abosede Adesida

**Affiliations:** 1 Department of Medical Microbiology and Parasitology, College of Medicine, University of Lagos, Lagos, Nigeria; 2 Molecular Biology and Biotechnology Division, Nigerian Institute of Medical Research, Yaba, Lagos, Nigeria; 3 Department of Microbiology, Faculty of Science, University of Lagos, Akoka, Lagos, Nigeria

**Keywords:** Prevalence, plasmid, salmonellosis, children

## Abstract

**Introduction:**

Non-typhoidal Salmonella is the causative agent of gastroenteritis, a food-borne and zoonotic infection which is a major cause of high morbidity and death among children under 5 years of age especially from resource poor settings like the developing countries.

**Methods:**

This study was carried out for 6 months to determine the prevalence and plasmid profile of non-typhoidal salmonellosis in children in Lagos metropolis. A total of 105 stool samples were collected from diarrheal children aged 3 months to 12 years and processed during this period. The isolates were identified using Selenite F Broth, Salmonella-Shigella Agar, Kligler Iron Agar, and Motility-indole-Urea medium, citrate and sugar utilization tests.

**Results:**

A total number of 127 isolates were identified, 2 of which are Salmonella enteritidis (1.6%). The non-typhoidal Salmonellae were sensitive to ciprofloxacin, cetotaxime, streptomycin, cotrimxazole and tetracycline. Only one of the 2 isolates (50%) was sensitive to amoxillin and sulphonamide while none of them (0%) was sensitive to cefuroxime.

**Conclusion:**

The plasmid analysis of the isolates showed that they harboured no detectable plasmids; this suggests that the resistance was chromosomally mediated.

## Introduction

Non-typhoidal salmonellosis, an infection caused by non-typhi *Salmonella* is transmitted either from animal to human or from human to human causing infection. The non-typhoid *Salmonella* species are freely present in the environment and reside in the gastrointestinal tracts of animals. A 2010 study of worldwide burden of non-typhoidal gastroenteritis estimated 2.5 million cases of the disease and 4100 deaths per year in Africa [1]. The mean infective dose to produce clinical or subclinical infection in human is 10^5^-10^8^ *Salmonellae* [2]. The non-typhoid *Salmonella* are grouped into *Salmonella typhimurium* and *Salmonella enteritidis* [3]. Salmonella typhimurium infects a wide range of host species, from animals to man and it is usually through contaminated foods while *Salmonella enteritidis* is widely distributed in domestic and wild animals, particularly rodents, and sporadic in man as a cause of food poisoning, it can be contacted from undercooked eggs [4]. In the 1970s it was estimated that 280,000 human cases of salmonellosis per year were associated with reptiles leading to the ban on turtles with carapace lengths less than 4 inches (i.e. would fit in a child's mouth), this led to a substantial reaction in paediatric cases of salmonellosis, but recent studies have suggested that sporadic human cases of Salmonella infection are still associated with reptiles [5, 6]. Children especially those under ten years of age are susceptible to severe salmonellosis after contact with reptiles [6].

Human cases of salmonellosis are acquired through direct contact with animals [6] and by ingestion of contaminated foods [4, 7]. This is because salmonellosis is a zoonosis and has an enormous animal's reservoir. It is generally contacted through the consumption of contaminated food of animal origin (mainly meat, shellfish, poultry, eggs and egg dishes and milk); high intake of dairy products (ice cream, cheese, milk) that are inadequately pasteurized or are contaminated with faeces can lead to salmonellosis because of the ability of *Salmonella* to survive in meats and animal products that are not thoroughly cooked [4]. In addition, contaminated water and vegetables contaminated from manure have been implicated in its transmission [8, 9]. It is transmitted through the ingestion of food or water contaminated with animal or human waste including contaminated raw vegetables or fruits [10, 11]. Transmission through the faecal-oral route is important, especially from persons who have diarrhoea or who are incontinent. Non- typhi *Salmonella* (NTS) infections are a frequent cause of self-limited diarrhoeal illness in healthy children. However, this can lead to bacteraemia in them if not properly handled, as a result of complication of NTS infection [8, 12]. In Africa and most other developing regions, multidrug resistance, particularly to commonly available antibiotics, remains a major challenge for the healthcare system [9, 13]. In particular, multidrug-resistant NTS have caused life-threatening invasive disease outbreaks in children in many African countries, including Nigeria [14–16]. The aim of this study was to determine the prevalence of non-typhoidal salmonellosis in children in Lagos metropolis, establish the antibiotic susceptibility pattern of the non-typhoidal *Salmonella* isolates and analyse the plasmid profile of the non-typhoidal *Salmonella* isolated.

## Methods

A total of 105 stool samples were collected from diarrhoeic children aged 3 months to 12 years over a period of 6 months. 46 samples were collected from Lagos State University Teaching Hospital, Ikeja. 19 samples were collected from the General Hospital, Gbagada while 11 samples were collected from the Lagos University Teaching Hospital, Idi-Araba. Also, 29 samples were collected from children from Olusosun area, Oregun.

### Isolation of Non-typhi *Salmonella*

A loopful of stool sample was inoculated aseptically into a test tube containing 5ml of Selenite F broth. This was incubated at 37oC for 24 hours after which the stool samples were subcultured from the Selenite F broth onto *Salmonella*-Shigella Agar (SSA). The plates were incubated at 37oC for 24 hours. Colonies suspected to be Salmonella species were selected from Salmonella-Shigella agar and subcultured onto SSA to achieve pure isolate cultures and transferred to vials of Tryptone Soy Broth (TSB) containing 20% glycerol. The broth was incubated at 37oC for 24hours and the organisms were maintained in the broth in the freezer at -20oC during storage.

### Presumptive identification tests

The suspected Salmonella isolates were subcultured onto SSA and incubated at 370C for 24 hours. Isolates were inoculated on Kligler Iron Agar (KIA) slope and Motility-Indole-Urea (MIU) medium and incubated overnight at 37oC. Urease test was done to rule out Proteus and indole test was used to distinguish between *Salmonella* species and other enterobacteria that can break down the amino acid tryptophan with the release of indole. The isolates were presumptively identified as *Salmonella* species by their reactions on KIA and MIU.

### Sugar utilization test (Peptone Water Sugars)

Presumptively identified non-typhi *Salmonella* were further tested using Sucrose, Arabinose, Xylose, and Mannitol. Sugar fermentation was confirmed by colour change from purple to yellow after incubation at 37oC for 48 hours.

### Citrate utilization test

Slopes of Simmon's citrate agar were streaked with the suspension of the organism and the butt was then stabbed using a sterile straight inoculating wire. The bottle was then incubated at 37oC for 48 hours. The ability of organism to utilize citrate was confirmed by colour change from green to blue. Non-typhi *Salmonella* utilizes citrate as opposed to *Salmonella typhi* which does not.

### Antimicrobial susceptibility testing

This was done using the disc diffusion method of National Committee for Clinical Laboratory Standards (NCCLS) [17]. About four well isolated colonies were emulsified in 3ml of sterile physiological saline; turbidity of the inoculum was compared with 0.5 Mcfarland standard and then swabbed on the surface of nutrient agar plates with sterile swab sticks. Gram-negative multidiscs of antimicrobials comprising of amoxicillin (25µg), tetracycline (30 µg), cefuroxime (25 µg), sulphonamide (30 µg), streptomycin (15 µg), cotrimoxazole (25 µg), ciprofloxacin (5 µg), and cefotaxime (30 µg) were placed on the inoculated plate using sterile forceps. The plates were then incubated at 370c for 24 hours. The zones of inhibition of the bacteria by the antimicrobials were measured in millimeters using a ruler, and compared with the zone of inhibition of the control organism used- *Escherichia coli* NCTC 148C.

### Plasmid profile

Plasmid DNA isolation was done using alkaline phosphate method of Birnboin and Doly [18]. The *Salmonella enteritidis* isolates were inoculated on Mueller Hinton agar and incubated at 370c for 24 hours. The bacteria were then harvested using wire loop and suspended in eppendorf tubes containing 200µl of buffer 1A (400mM Tris, 20 mM Na EDTA, Acetic acid to pH 8.0), then it was vortexed. Exactly 400 µl of lysing solution (4% Sodium Dodecyl Sulphate (SDS), 100mM Tris) was added to the washed cells and the tubes were inverted 20 times at room temperature. About 300 µl of ice-cold buffer 2B (3M Na acetate, Acetic acid to pH 5.5) was added and vortexed and kept on ice for 3 minutes. The lysates were then centrifuged at 3,000xg for 15 minutes. About 700 µl of chloroform was added and then vortexed. It was then centrifuged at 3,000xg for 10 minutes. To 500µl aqueous layer, 1ml of absolute ethanol was added and it was kept on ice for 1 hour, it was then centrifuged at 3,000xg for 30 minutes. Pellets were washed with 70% ethanol and centrifuged at 3,000xg for 15 minutes. The pellets were dried and 100 µl of buffer 3C (10mM Tris, 2mM Na2 EDTA, Acetic acid to pH 8.0) was added. 20 µl from the resulting solution was added to 5µl bromophenol blue (which serves as a tracking dye). λ DNA Hind 111 Digest marker of known molecular weight was also prepared. It was then introduced into the wells in the electrophoretic tank containing agarose gel stained with 0.05% ethidium bromide (intercalating agent), starting with the marker. Electrophoresis was run at 70V for 1 hour, 30 minutes. The gel was viewed under the Ultraviolet (UV) light, DNA bands were visualized with a UV transilluminator (UVP) and the migration distances of the samples were then compared with the migration distances of the λ DNA.

## Results

**Figure 1:**
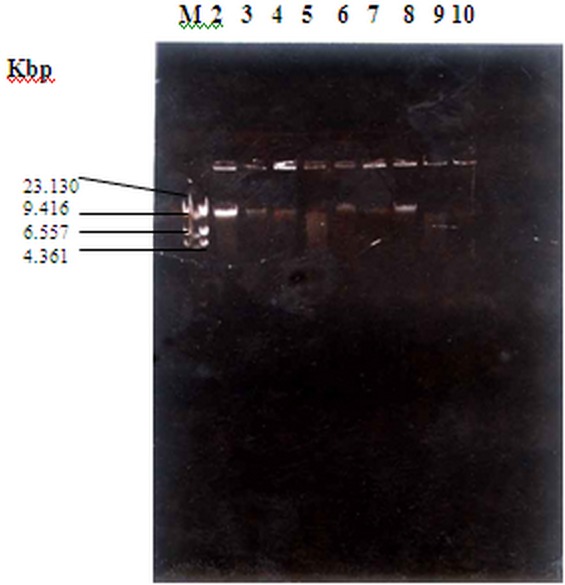
Agarose gel electrophoresis showing plasmid profile of Salmonella enteritidis

Out of the 105 samples processed, 96 samples (91.4%) yielded bacterial growth while 9 samples (8.6%) yielded no bacterial growth. One hundred and twenty nine isolates were recovered from the stool samples. These comprised 2 *Salmonella enteritidis* (1.6%), 33 *Escherichia coli* (26%), 13 *Enterobacter species* (10.2%), 36 Proteus species (28.3%), 22 *Pseudomonas species* (17.3%), and 21 *Klebsiella species* (16.5%) (Table 1). The isolates were identified using standard methods as described by Cowan and Steel, (1993) (Table 2 and Table 3). Table 4 shows the antibiotic sensitivity pattern of the isolates. The 2 *Salmonella enteritidis* isolates (100%) were sensitive to Ciprofloxacin, Cefotaxime, Streptomycin, Cotrimoxazole and Tetracycline. Only one of the 2 isolates (50%) was sensitive to Amoxicillin and Sulphonamide while none of them (0%) was sensitive to Cefuroxime. Figure 1 and Table 5 show the plasmid analysis of the *Salmonella species*. The 2 *Salmonella enteritidis* harboured no plasmid. The analysis showed the chromosomal DNA of the isolates, and both the chromosomal DNA and the plasmid of the molecular weight marker (λ Hind111 Digest) ranging from 4.361 to 23.130 kilobase pairs (kbp).

**Table 1:** Frequency of isolates recovered from children's stool samples

ISOLATE	NUMBER	PERCENTAGE
*Escherichia coli*	33	26%
*Enterobacter species*	13	10.2%
*Proteus species*	36	28.30%
*Pseudomonas species*	22	17.30%
*Salmonella* *enteritidis*	2	1.60%
*Klebsiella species*	21	16.50%
Total	127	100.00%

**Table 2:** Biochemical test pattern of isolated *Salmonella* enteritidis and *Escherichia coli*

ISOLATE	KIA				MIU			SIMMONS	GRAM
CODE	SLOPE	BUTT	H_2_S	GAS	M	I	U	CITRATE	STAIN
S26	Y	Y	-	+	+	+	-	-	-
S34	Y	Y	-	+	+	+	-	-	-
S63	R	Y	+	+	+	-	-	+	-
S71	Y	Y	-	+	+	+	-	-	-
S78	Y	Y	-	+	+	+	-	-	-
S92	R	Y	+	+	+	-	-	+	-
S95	Y	Y	-	+	+	+	-	-	-
SIO4	Y	Y	-	+	+	+	-	-	-

**Key**: Y-Yellow (acid reaction)

R-Red (alkaline reaction)

+ Positive reaction

-Negative reaction

**Table 3:** Sugar utilization tests of *Salmonella enteritidis* isolated

ISOLATE CODE	ARABINOSE	MANNITOL	SUCROSE	XYLOSE
S63	+	+	-	+
S92	+	+	-	+

**Table 4:** Antibiotic sensitivity pattern of *Salmonella enteritidis* and other isolates

ISOLATE	AMP	TET	CRX	SUL	STR	COT	CPR	CTX
*Salmonella enteritidis* S63	R	S	R	R	S	S	S	S
*Salmonella enteritidis* S92	S	S	R	S	S	S	S	S
*Escherichia coli* S26	R	S	R	S	S	R	S	S
*Escherichia coli* S34	S	R	S	R	R	R	S	S
*Escherichia coli* S71	R	R	R	S	R	S	S	R
*Escherichia coli* S78	R	R	R	R	S	R	S	S
*Escherichia coli* S95	R	R	S	S	R	S	S	R
*Escherichia coli* S104	R	S	R	R	S	S	S	S
*Escherichia coli* NCTC 148C	S	S	R	R	S	S	S	S

**Key**: AMP = Amoxycillin; TET = Tetracyclin; CRX = Cefuroxime; SUL = Sulphonamide; STR = Streptomycin; COT = Cotrimoxazole; CPR = Ciprofloxacin; CEF = Cefotaxime; R = Resistant (0-10mm) S = Sensitive (11-29mm); NCTC: National Collection Type Culture

**Table 5:** Plasmid profile of non-typhi Salmonella

N	Sample	Isolate	No Of Plasmids	Molecular Weight
				(Kilobase pairs)
1	Marker	*-*	4	4.361 - 23.130
2	S26	*Escherichia coli*	None	None
3	S104	*Escherichia coli*	None	None
4	S34	*Escherichia coli*	None	None
5	S63	*Salmonella* enteritidis	None	None
6	S104	*Escherichia coli*	None	None
7	S78	*Escherichia coli*	None	None
8	S71	*Escherichia coli*	None	None
9	S92	*Salmonella* *enteritidis*	None	None
10	S95	*Escherichia coli*	None	None

## Discussion

This study shows the isolation of non-typhi *Salmonella* from children in Lagos. Non-typhi *Salmonella* in particular, *Salmonella enteritidis* was isolated. It was reported that non-typhi Salmonella are common cause of diarrhoea in children less than 5 years of age [19, 20], these studies corroborates this view because the non-typhi Salmonella isolated were from stool samples of children aged 7 months and 2 1/2 years. Non- typhi *Salmonella* (NTS) are a frequent cause of self-limiting diarrhoeal illness in healthy children, infections are acquired as food poisoning and are usually self-limiting, and antimicrobial treatment is not recommended for uncomplicated illnesses [16, 21], this view reflects in this study in that the infection subsides on its own making isolation of the implicated bacterium to be difficult. The low rate of isolation of non-typhi *Salmonella* in this study could also be due to intake of self-prescribed antibiotics administered by mothers before bringing the children to the hospitals; this is a common practice as a first line treatment before taking children to the hospital. Meats and animals products that are not thoroughly cooked, animal products, poultry or poultry derived products are the major mode of transmission for non-typhoidal *Salmonellae* because of the ability of *Salmonella* to survive in them [4, 10]. In this part of the world, proper cooking of our food products before consumption is the usual practice. This can destroy this pathogen, hereby reducing the isolation rate. The 2 strains (100%) of *Salmonella enteritidis* isolated were sensitive to ciprofloxacin, cefotaxime, streptomycin, cotrimoxazole and tetracycline. Only one of the 2 isolates (50%) was sensitive to amoxicillin and sulphonamide while none of them (0%) was sensitive to cefuroxime. Resistance of the *Salmonella* isolates to cefuroxime, a second generation cephalosporin agrees with [14, 22], because these pathogens developed resistance over time due to abuse and misuse of drugs by practicing self-medication. The resistance to amoxicillin agrees with earlier reports by Ibrahim et al [23] and Gordon et al [16].

It has been reported that *Salmonella species* have become progressively more resistant to clinically useful antibiotics in Africa and most other developing regions, multidrug resistance, particularly to commonly available antibiotics, this remains a major challenge for the healthcare system and multidrug-resistant NTS have caused life-threatening invasive disease outbreaks in many African countries, including Nigeria [24, 25]. The result of this study corroborates this view. In this study, the isolates were sensitive to cefotaxime, a third generation cephalosporin. This study agrees with the submission of [20, 26] and guidelines on management of paediatric illness [27] that the third generation cephalosporins are effective for the treatment of non-typhoidal salmonellosis in children. In this study, the *Salmonellae* isolates harboured no detectable plasmids; this suggests that the resistance was chromosomally mediated. Non-typhoidal *Salmonella* infection Surveillance Protocol [28] agrees with study, showing that salmonellosis outbreak plays an important role in isolation of non typhi *Salmonella*. There was no outbreak of non typhoidal salmonellosis during the course of this study. In addition, the self-limiting nature of this infection reflects in the number of isolates of non-typhoidal *Salmonella species* isolated, because it might have subsided before laboratory investigation. In this study, 2 isolates of non-typhoidal *Salmonella: Salmonella enteritidis* were recovered giving 1.6% isolation rate. The isolates were from stool samples of children aged below five years showing the predisposition of children to non-typhoidal salmonellosis.

## Conclusion

There was no outbreak of non-typhoidal salmonellosis during the course of this study, as shown by the isolation rate. Also, the isolates did not harbour plasmids suggesting that resistance was chromosomally mediated. Furthermore, third generation cephalosporins like Cefotaxime are the drugs of choice for the treatment of non-typhoidal salmonellosis in children in Lagos metropolis.
